# Evaluation of the potential of a new ribavirin analog impairing the dissemination of ovarian cancer cells

**DOI:** 10.1371/journal.pone.0225860

**Published:** 2019-12-11

**Authors:** Anaïs Wambecke, Carine Laurent-Issartel, Johanne Leroy-Dudal, Florence Giffard, Fanny Cosson, Nadège Lubin-Germain, Jacques Uziel, Sabrina Kellouche, Franck Carreiras

**Affiliations:** 1 Equipe de Recherche sur les Relations Matrice Extracellulaire-Cellules, ERRMECe (EA1391), Institut des Matériaux, I-MAT (FD4122), University of Cergy-Pontoise, MIR, rue Descartes, France; 2 Normandie University, UNICAEN, INSERM U1086 ANTICIPE (Interdisciplinary Research Unit for Cancers Prevention and Treatment, BioTICLA Axis (Biology and Innovative Therapeutics for Ovarian Cancers), Esplanade de la Paix, Caen, France; 3 Laboratoire de Chimie Biologique, University of Cergy-Pontoise, mail Gay-Lussac, Cergy-pontoise, France; Universite de Technologie de Compiegne, FRANCE

## Abstract

Epithelial ovarian cancers are insidious pathologies that give a poor prognosis due to their late discovery and the increasing emergence of chemoresistance. Development of small pharmacological anticancer molecules remains a major challenge. Ribavirin, usually used in the treatment of hepatitis C virus infections and more recently few cancers, has been a suggestion. However, Ribavirin has many side-effects, suggesting that the synthesis of analogs might be more appropriate.

We have investigated the effect of a Ribavirin analog, SRO-91, on cancer cell behavioral characteristics considered as some of the hallmarks of cancer. Two human ovarian adenocarcinoma cell lines (SKOV3 and IGROV1) and normal cells (mesothelial and fibroblasts) have been used to compare the effects of SRO-91 with those of Ribavirin on cell behavior underlying tumor cell dissemination. SRO-91, like Ribavirin, inhibits proliferation, migration, clonogenicity and spheroids formation of cancer cells. Unlike Ribavirin, SRO-91 is preferentially toxic to cancer compared with normal cells. An *in vitro* physiologically relevant model showed that SRO-91, like Ribavirin or cisplatin, inhibits cancer cell implantation onto peritoneal mesothelium. In conclusion, SRO-91 analog effects on tumor dissemination and its safety regarding non-cancerous (normal) cells are encouraging findings a promising drug for the treatment of ovarian cancer.

## Introduction

Ovarian cancer is the gynecological malignancy with the highest case-to-mortality ratio in the western world. Because ovarian cancer is often asymptomatic, it is generally diagnosed at an advanced stage, giving a poor prognosis [1]. Although the majority of tumors initially respond to standard treatments combining surgery and platinum-based chemotherapy, frequent recurrence and subsequent acquired chemoresistance, as also widespread dissemination, are responsible for the therapeutic ineptness, leading to an overall 5-year survival rate of 40% [2]. In this context, new drugs or therapeutic strategies are needed, in particular, in finding novel cytotoxic systems or molecules that can specifically target malignant cells while sparing healthy cells.

In about 90% of cases, ovarian cancers arise from the transformation of the ovarian surface epithelium. Cells proliferate and spread prevalently by direct extension into adjacent tissues and by cancer cells exfoliating from the primary tumor into the peritoneal cavity. Thus, ovarian cancer cells are preferentially found as a solid tumor mass adhering to the ovary, as multicellular aggregates in the abdominal cavity (referred to as spheroids), and as cells adhering to and invading the peritoneal mesothelium [3,4]. The mesothelium, a single layer of flat cells covering the peritoneal cavity and its organs, is the first barrier met by ovarian tumor cells and is the major site of ovarian carcinoma metastasis before invading the underlying connective tissue rich in fibroblasts.

**Fig 1:**
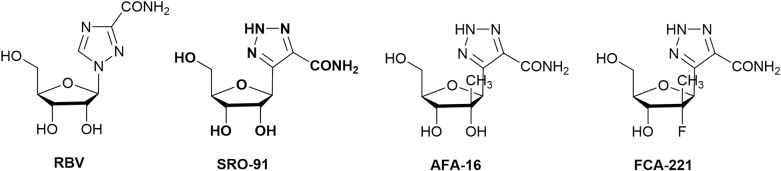
Structure of ribavirin (RBV), SRO-91, AFA-16 and FCA-221 analogs.

Considering these data, the development of small pharmacological molecules that can interfere with the molecular mechanisms conducive to the survival of cancer cells presents a major challenge. Among these molecules, ribavirin (RBV) has been suggested as useful in anti-cancer therapy [5,6] (Fig 1).

Ribavirin (1-β-d-ribofuranosyl-1,2,4-triazole-3-carboxamide) is a synthetic nucleoside well known to be a broad-spectrum anti-viral agent, and is extensively used in the treatment of hepatitis C infections and Respiratory Synsivial Virus (RSV). RBV may act through pleiotropic mechanisms as it: i) modulates immunity, inducing a shift from a Th2 to a Th1 cytokine profile *in vivo*, and in mast cell mediator release, stimulated by IgE; ii) inhibits inosine monophosphate dehydrogenase (IMPDH) activity that is a key enzymic reaction in guanosine biosynthesis involved in viral replication; and iii) decreases RSV-mediated NFκB activation [7]. Ribavirin is already known to be effective in cancer therapy; it targets an oncogene, the eukaryotic translation initiation factor eIF4E, elevated in approximately 30% of cancers, including many leukemias and lymphomas, breast, esophagea, and ovarian cancers. Phosphorylation of eIF4E contributes to cell transformation and increased export of a subset of growth-promoting mRNAs, such as cyclin D1 and c-Myc [8]. RBV is a potent anticancer agent, being a strong inducer of apoptosis and a moderate inducer of differentiation in leukemic cells [7]. It also reduces cell proliferation and suppresses clonogenic potential of breast cancer cells, correlating with reduced mRNA export and protein expression of important eIF4E targets [9]. RBV also has anti-proliferative and cytotoxic effects on ovarian cancer (OVCAR-5) cells [7,10]. RBV treatment in clinical trial led to substantial clinical benefit in patients with a poor prognosis in acute myeloid leukemia (AML) [11]. Besides the cost, treatment with RBV has a number of side effects, including hemolytic anemia.

Reduction in toxicity and the side effects of RBV was then a goal, and a large number of RBV analogs were synthesized, such as carba-1,2,3-triazoles, 1,2,4-triazoles, pyrazoles and tetrazoles. Triazole is a structural unit of interest in medicinal chemistry as it is isosteric in the peptide bond and is an aromatic nucleobase analog. Therefore, we have described the synthesis of ribosyl triazoles based on a *C*-alkynylation reaction of ribose, followed by a 1,3-dipolar cycloaddition [12]. A series of RBV analogs were synthesized, our interest being particularly focused on SRO-91 [13] (Fig 1). This compound, bearing a carbon-carbon bond between ribose and triazole, unlike ribavirin, has antiviral activity against HCV similar to this one (unpublished results). However, no studies on the anti-cancer effect of SRO-91 have been conducted to date. Considering these arguments, we have focused on the impact of SRO-91 and two other 2’-Me-*C*-nucleoside analogs AFA-16 and FCA-221 on ovarian cancer [14] (Fig 1), and their behavior that underlies dissemination, considered as hallmarks of malignant cancers (proliferation, resisting cell death, invasiveness, etc.) [15]. We have paid particular attention on the potential safety of RBV analogs regarding non-cancerous (normal) tissue cells. Inhibitory effects of SRO-91 were found on the different hallmarks of ovarian cancer dissemination. The relevance to the use of SRO-91 as an anticancer treatment is reinforced it relative non-toxicity to non-cancerous cells that were tested.

## Materials and methods

### Cells and reagents

The human ovarian adenocarcinoma cell line, IGROV1, was kindly provided by Dr. J. Bénard, and SKOV3 cell lines were kindly supplied by Dr L. Poulain from the BioTICLA team INSERM U1086 (Biologie et Thérapies Innovantes des Cancers Localement Agressifs, Caen, France). The cells were grown in RPMI-1640 glutaMAX (Life technologies, France) containing 0.07% (v/v) sodium bicarbonate supplemented with 10% fetal bovine serum (FBS, Biosera, France) in a humidified air atmosphere with 5% CO_2_ in air at 37°C. The 2 well characterized human ovarian cancer cell lines, IGROV1 and SKOV3, were used for their ability to mimic the progression of ovarian carcinoma when injected *in vivo* in mouse models [16]. The human mesothelial cell line, MeT-5A, was purchased from American Type Culture Collection (ATCC, Manassas, Va., USA). The cells were grown in M199 supplemented with 3.3 nM epidermal growth factor (EGF; Sigma St-Quentin-Fallavier, France), 870 nM insulin, 400 nM hydrocortisone (Sigma), 1.25 g/l sodium bicarbonate (Invitrogen) and 10% FBS. Human fibroblasts BJ cells purchased from ATCC (France), with a certificate of conformity, were cultured in DMEM (Life technologies, France) supplemented with 10% FBS. All cells were cultured in humidified atmosphere of 5% CO_2_ in air at 37°C. Subconfluent cells were harvested with 0.25% trypsin-EDTA (Life technologies, France). For the experiments, cells were exposed to a range of concentrations of RBV or its analogs.

Lyophilized Ribavirin (Virazol®) was purchased from TCI EUROPE NV (Zwijndrecht, Pays Bas). SRO-91, AFA-16 and FCA-221 were synthesized as described by Solarte et al. and Cosson et al. [13,14]. The chemotherapeutic drug, cisplatin (*cis*-diammine-dichloroplatinum II) was purchased from Mylan (Saint-Priest, France)—cisplatin being the gold standard drug in the treatment of ovarian cancer.

### Ascites samples

The ascites samples were collected at the Cancer Center F. Baclesse (Caen, France), were certified cytologically as positive for malignant cells, and stored in the OvaRessources Biological Resources Center (BRC) (NF-S 96900 quality management, AFNOR N°2016:72860.5). The medical officer and the scientific officer of the BRC are respectively Dr Cécile Blanc Fournier and Dr Laurent Poulain. All biological collection from BRC was declared by the project Manager of the Clinical Research Department (Alexandra Leconte) to the MESR (Ministry of Education, Health and Research, France, N°DC-2010-1243 and amendments DC-2013-1849, DC-2016-2641 and DC-2017-2923). The date of the favourable opinion on the constitution of the collection is 17 of September 2011. We obtained written informed consent from the patient. The study was approved by the ethical committee “CPP North-West III” (Committee for the Protection of Persons).

Ascites samples from a 73-year-old patient with a papillary serous adenocarcinoma, stage IIIb, grade 3 were collected in May 2014. Immediately after collection, the samples were centrifuged at 400g for 5 min and the supernatants stored at -20°C. The samples were sterilized by filtration (0.2μm, low-binding) and supplemented with penicillin (200 UI/mL)/ streptomycin (200 μg/mL) before cell culturing.

### Cell proliferation and viability assay

In the proliferation assay, IGROV1, SKOV3, Met-5A or Fibroblasts BJ cells were seeded in 24-well plates at 10,000/cm^2^ and exposed to ribavirin analogs or ribavirin at 0, 20 or 50 μg/mL for 7 days of culture. The number of viable cells was measured daily by the trypan-blue exclusion test. Results are representative of 3 or more independent experiments done in triplicate, and are expressed as mean ± standard deviation.

### Western blotting

Cell homogenates were prepared using lysis buffer. The lysates were centrifuged at 12,000 g for 10 min at 4°C and the protein concentration of the supernatant determined by the Bradford method (Bio-Rad, Life Science Group, Marnes-la-Coquette, France). Equal amounts of protein (20 μg) were resolved using denaturing 9% sodium dodecylsulfate–polyacrylamide gel before being Western blotted using anti-PARP and cleaved PARP (9542, Cell signaling, Danvers, MA, USA) or anti-actin (A5441, Sigma-Aldrich) as a loading control. An Immobilon substrate kit (Millipore) was used for detection of bands.

### BrDU incorporation

For the BrDU assay, cancer cells were seeded on to glass coverslips in 24-well plates at 10,000/cm^2^ and exposed to SRO-91 or ribavirin at 0, 20 or 50 μg/mL for 7 days of culture. After each time-point, the medium was supplemented with BrDU (40 μM, B9285, Sigma) for 3 h before the cells were washed with PBS. They were fixed with 3% paraformaldehyde and incubated for 30 min with HCl (2N) (LCH, Chimie, Les Aires France) to denature DNA. Cells were incubated with borate salt (0.1 M, Sigma) to neutralize HCl before being permeabilized with 0.1% Triton X100 in PBS. Cells were incubated for 2 h at room temperature with anti-BrDU (B2531, Sigma) antibody. After washing, the coverslips were incubated with fluorescent secondary antibodies Alexa Fluor 555-conjugated anti-mouse antibody (A21424, Invitrogen). Cell nuclei were stained with DAPI (4,6-diamidino-2-phenylindole dihydrochloride, D9542, Sigma Aldrich). Controls, in which primary antibodies were replaced with PBS, were negative. Coverslips were mounted in Prolong Gold antifade Reagent and examined with laser scanning confocal microscopy with a mosaic of 4x4 independent fields. Positive BrDU (cells/field) were quantified with the software ImageJ ® (National Institutes of Health, Bethesda, MD).

### Immunofluorescent staining

Cells were seeded on to glass coverslips in 24-well plates at 10,000/cm^2^ and exposed to different concentrations of ribavirin analogs or ribavirin (20 or 50 μg/mL) for 1, 3 and 7 days of culture. At each time-point, cells were fixed with 3% paraformaldehyde (PFA) in PBS and washed with PBS containing 0.5% BSA. They were permeabilized with 0.1% Triton X100 in PBS and incubated for 2 h at room temperature with anti-αv integrins (sc-9969, Santa-Cruz) or anti-eIF4E (610270, BD PharMingen) antibodies. After washing, the coverslips were incubated with the appropriate fluorescent secondary antibodies: Alexa Fluor 555-conjugated anti-mouse antibody (A21424, Invitrogen) or Alexa Fluor 488-conjugated anti-mouse antibody (BD Transduction Laboratories). The actin cytoskeleton was stained with FITC- or TRITC-phalloidin (P5282 or P1981, Sigma Aldrich). For each assay, cell nuclei were stained with DAPI (4,6-diamidino-2-phenylindole dihydrochloride, D9542, Sigma Aldrich). Coverslips were mounted in Prolong-Gold Antifade Reagent (P36930, Invitrogen) and examined by laser scanning confocal microscopy (LSM710, Zeiss). Controls, in which primary antibodies were replaced with PBS, were negative. Fluorescence microscopy figures were processed using Fiji software [17].

### Analysis of the nuclear area

Cells were seeded on to glass coverslips in 24-well plates at 10,000/cm^2^ as described above. At each time-point, cells were fixed with 3% PFA in PBS and washed with PBS containing 0.5% BSA. Cell nuclei were stained with DAPI. Coverslips were mounted in Prolong-Gold Antifade Reagent and examined by laser scanning confocal microscopy. The average area of the nuclei in μm^2^—according to the different conditions over time—was measured using Image J^®^ software (National Institutes of Health, Bethesda, MD, USA).

### Cell migration

Ovarian cancer cells were seeded in 24-well plates and grown until confluent. Monolayers were scratched and migration continued thereafter in SRO-91 or RBV (50 μg/mL). Cell migration was monitored by time-lapse microscopy (inverted microscope Leica DMI6000 B) for 24 h at 30 min intervals. The percentage of cell migration in 3 independent experiments was quantified using ImageJ software.

### Clonogenicity assay

Cancer cells were seeded in 6-well plates at 500 cells/well (to obtain isolated cells) and incubated in the presence of SRO-91 or RBV (20, 50 μg/mL) for 7 days as described by Carduner et al. [18]. The colony-forming ability of the cells was followed daily by phase-contrast microscopy. Pictures are representative of at least 5 fields of 3 independent experiments.

### Spheroid formation

The method of generating multicellular aggregates, spheroids, has previously been described [19]. Briefly, to prevent cell adhesion to a substratum, culture flasks (25 cm^2^) were filled with 4 mL 1% agarose (Eurobio, Courtaboeuf, France) in serum-free medium and allowed to solidify for 30 min at room temperature. Subconfluent IGROV1 cells were collected with trypsin–EDTA and resuspended in complete medium supplemented with SRO-91 or RBV (20, 50 μg/mL) at 1.5x10^6^ cells/flask. Multicellular aggregates were cultivated for 7 days and followed each day by phase-contrast microscopy. The number of multicellular aggregates was counted in at least 10 fields in each condition. Results are representative of at least 3 independent experiments.

### Co-culture model and cell-cell adhesion assay

To mimic cancer cell dissemination on to a mesothelium, a co-culture assay of ovarian cancer cells and healthy mesothelial cells was used. Briefly, mesothelial cells were first grown on glass coverslips in 24-well plates until confluence in complete M199 medium. Sub-confluent cancer cells were resuspended in RPMI-1640 without FCS and labeled with 5 μl/ml of Vybrant DiI solution (Invitrogen) by incubation for 15 min at 37°C. The DiI-labeled cells were washed 3 times and resuspended in M199-free FCS medium or ascites supplemented RBV (50 μg/ ml), SRO-91 (20, 50 μg/ ml) or Cisplatin (2.5 μg/ ml). Cancer cells (20,000/well) were added directly on to the mesothelial monolayer and incubated for 1 h at 37°C. Co-cultures of mesothelial cells grown as a monolayer and DiI-labeled tumor cells were observed using a Nomarski filter to ensure mesothelial integrity. Cells were fixed with 3% PFA and nuclei stained with DAPI. Coverslips were mounted in Prolong Gold antifade Reagent and examined by laser scanning confocal microscopy with a mosaic of 3x3 independent fields. Cancer cell adhesion to the mesothelial monolayer was determined by counting fluorescent cells on the underlying non-fluorescent mesothelial cells with the software ImageJ ®.

### Statistical analyses

All figures were obtained from at least 3 independent experiments carried out in triplicate. Data are expressed as mean ± SD. Nuclear surface area and co-culture assay were analyzed with unpaired Student's t-test (*** P<0.001, ** P<0.01, * P<0.05). All other reported comparison between control and compound treatment were performed by paired one way ANOVA test (* P<0.05, ** P<0.01, *** P<0.001).

## Results

RBV and its analogs (SRO-91, AFA-16 or FCA-221) were tested on several cancer cell behaviors underlying different steps of ovarian carcinoma dissemination. Thus, cell proliferation, colony formation (clonogenicity), cell migration, spheroid formation and cell implantation on the peritoneal mesothelium were estimated when treated with these molecules.

### SRO-91 or Ribavirin inhibits proliferation of ovarian cancer cells

**Fig 2:**
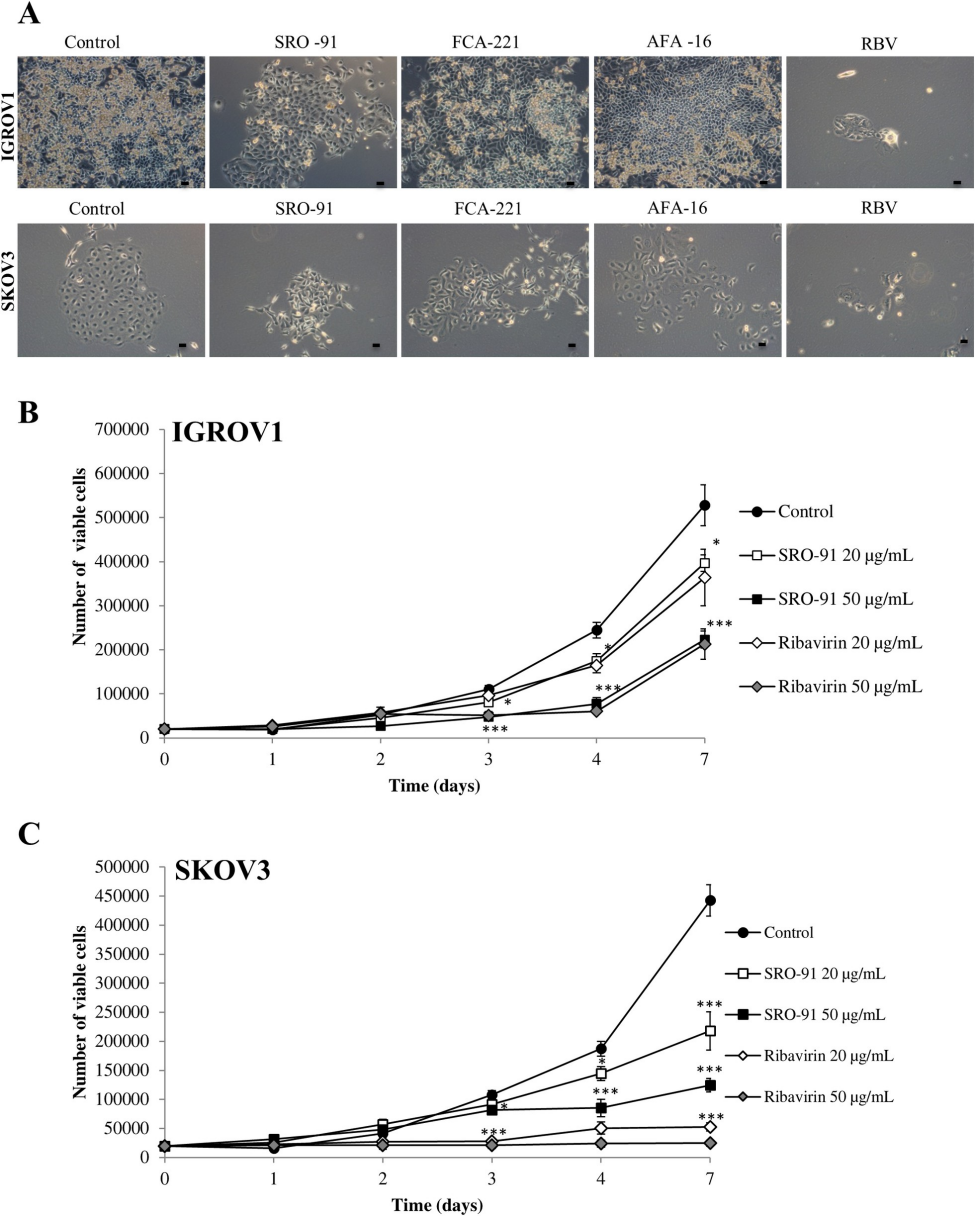
Viability of cancer cells after SRO-91 or ribavirin treatment. IGROV1 and SKOV3 were exposed to ribavirin analogs SRO-91, AFA-16, FCA-221 or ribavirin from 0 to 50 μg/mL for 7 days of culture. (A) Representative light micrographs were taken on day 7 (scale bar 50 μm). The number of viable (B) IGROV1 or (C) SKOV3 cells was measured daily by the trypan-blue exclusion test. Results are representative of 3 or more independent experiments done in triplicate, and are expressed as mean ± standard deviation. The inhibition of viable cells was significant from 3 days in both cells lines. For IGROV1 cells, Ctrl vs. SRO-91 or RBV 20 μg/ml (*P<0.05), Ctrl vs. SRO-91 or RBV 50 μg/ml (*** P<0.001). For SKOV3 cells, Ctrl vs. SRO-91 20 or 50μg/ml (*P<0.05) Ctrl vs.RBV (*** P<0.001).

IGROV1 and SKOV3 cell proliferation were estimated after treatment with SRO-91, AFA-16, FCA-221 or RBV. In these experiments, cells were exposed to different concentrations of drugs (from 0 to 50 μg/mL) for 7 days and the number of viable cells measured daily by the trypan-blue exclusion test. No inhibitory effect of AFA-16 or FCA-221 was seen on the growth of both cancer cells (Fig 2A). However, SRO-91 or RBV significantly inhibited the proliferation of IGROV1 and SKOV3 cells in a dose- and time-dependent manner **(**Fig 2B and 2C, respectively). In addition, doubling time of IGROV1 and SKOV3 cells, which is usually of 20 h and 30 h respectively, was extended after SRO-91 or RBV treatment to 33h and 43h for IGROV1 and 53h and > 96h for SKOV3, respectively. Inhibition of IGROV1 and SKOV3 cells began after 3 days of culture and was maintained until 7 days, with IC50 values of 23 μg/ml and 19 μg/ml for SRO-91, and 16 μg/ml and 10 μg/ml for RBV, respectively. The effect of RBV was notably more marked than SRO-91, and SKOV3 cells seem to be more sensitive to both molecules.

**Fig 3:**
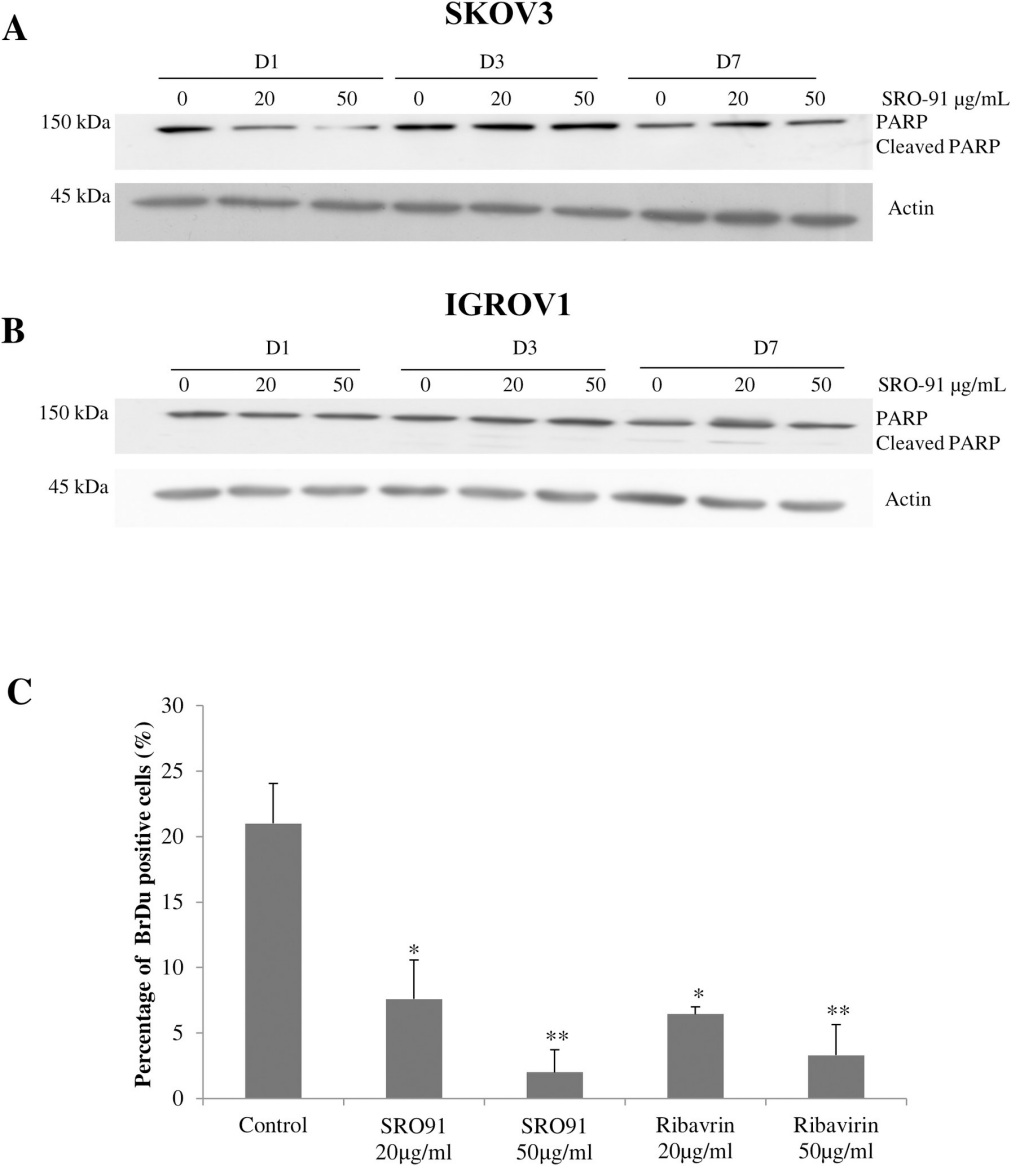
Expression of full length-/cleaved-PARP and S-phase analysis of cancer cells after SRO-91 treatment. Representative western blots for PARP and cleaved-PARP proteins, from SKOV3 (A) or IGROV1 (B) cells. Actin was used as a loading control. (C) Analysis of cell BrDU incorporation. Positive BrDU cells /fields after SRO-91 or RBV treatment of cancerous cells were quantified. P-values were calculated by Paired one-way ANOVA test (*P<0.05, **P<0.01).

Interestingly, inhibition of cell proliferation was not associated with cell death as estimated by the trypan-blue exclusion test. Since PARP cleavage is a sign of apoptosis, it was followed by western blot (Fig 3). PARP protein was expressed in the 2 cell lines whatever the culture conditions. PARP cleavage did not occur over the time after SKOV3 cell (Fig 3A, S1 Fig) treatment with SRO-91, where it was very slight with IGROV1 cells (Fig 3B, S2 Fig) from 3 days culture, even in control condition, probably due to their capacity to form spontaneously non-adherent multicellular clusters [20]. Thus, no or very weak apoptosis occurred in SKOV3 and IGROV1 cell under treatment. These results were consistent with the cell cycle analysis of cancer cell lines where few cells are in SubG1 peak (cell debris and apoptotic cells) independently to the treatment (S3 Fig).

To quantify cancer cells in S phase, BrdU incorporated into DNA was analyzed in the presence or absence of SRO-91 or RBV. The 2 compounds inhibited the percentage cells incorporating BrdU from day one in dose-dependent manner (Fig 3C), which correlates with an inhibition of proliferation. Cell cycle analysis showed no apparent blockage in the phases of the cell cycle, particularly S phase, but confirmed the decrease in the overall cell density upon treatment (S3 Fig).

Proliferation inhibition was not associated with cell death, but is probably due to a slow-down in the entry of cells into S-phase, which could explain the increase in the doubling time of ovarian cancer cells under treatment.

### SRO-91 affects slightly cell morphology of cancer cells

**Fig 4:**
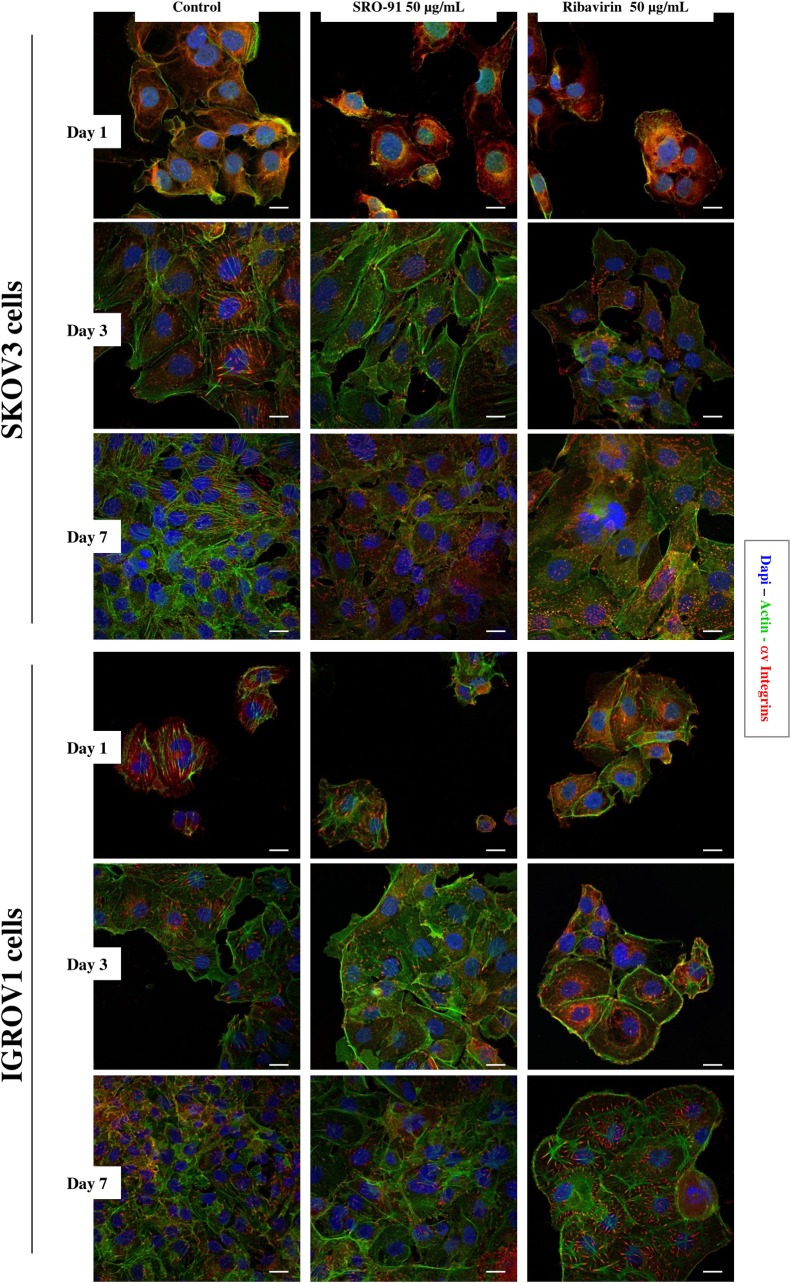
Cancer cells adhesive behavior and morphology. Immunofluorescent staining of actin cytoskeleton and αv integrins expressed by cancer SKOV3 and IGROV1 cells after SRO-91 or ribavirin treatment or without treatment (control). Cell nuclei were stained with DAPI. Staining was examined with laser scanning confocal microscopy. Scale bar is 30 μm.

**Fig 5:**
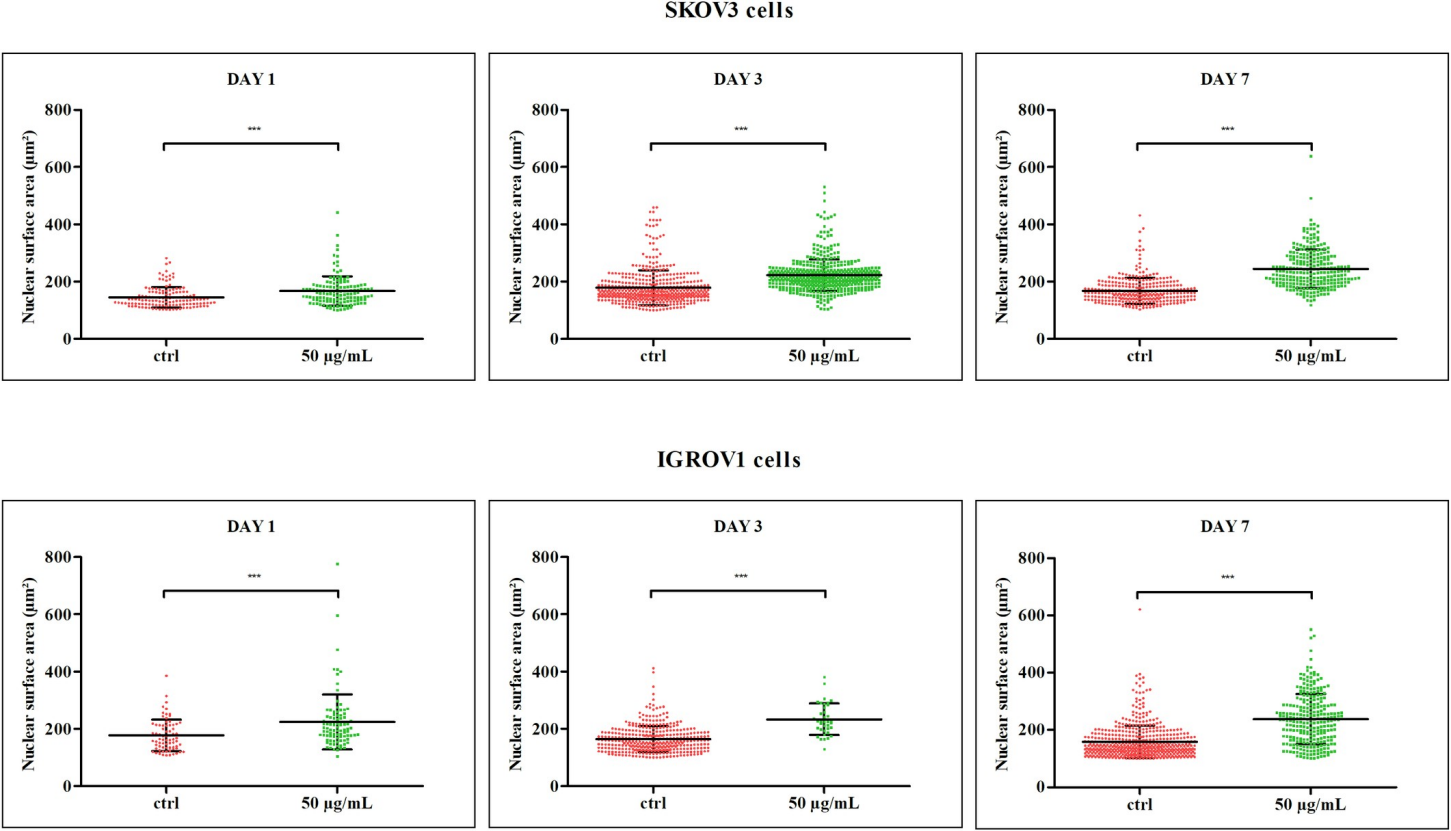
Analysis of the nuclear area. The average areas of the nuclei in μm^2^ after SRO-91 (50 μg/ml) treatment of cancerous cells was quantified. P-values were calculated by the unpaired t-test (*** P<0.001).

The effect of SRO-91 or RBV on cancer cell cytoskeletal morphology and adherence was also estimated by staining actin and αv integrins, which also involved structure, motility and cell-cell adhesion regarding the extracellular matrix. Slight modifications were seen between control culture and treated cells. Classical stress fibers, visualized in control conditions, shifted to more obvious filamentous actin organization and cortical pattern in treated cells (Fig 4). αv integrins forming adhesion structures as indents in staining were localized at the cell membrane, but showed differences in the intensity, structure (dashes/dots), distribution and organization in treated cells. The lack of massive rearrangement of the αv integrins and cytoskeleton after SRO-91/ RBV treatment was associated to an unaltered adhesive behavior as evidenced by cell adhesion assays (S4 Fig). The effect of SRO-91 or RBV treatment on vitronectin expression, main receptor of αv integrins, or laminin another matrix protein, did not act on the organization of the extracellular matrix of ovarian cancer cells (S5 Fig). We noted that no nuclear fragmentation occurred after treatment, confirming the absence of apoptosis. Interestingly, nuclear area increased in a dose- and time-dependent manner with SRO-91 or RBV treatment (Fig 5). An analysis of the relative size of the cells that depends on the shape of the cell and the nucleus was followed by flow cytometry with the forward scatter (FS) parameter. Cell size and nuclear shape were positively correlated because FS parameter increased with the treatment (S6 Fig).

### SRO-91 and RBV reduce cancer cell migration

**Fig 6:**
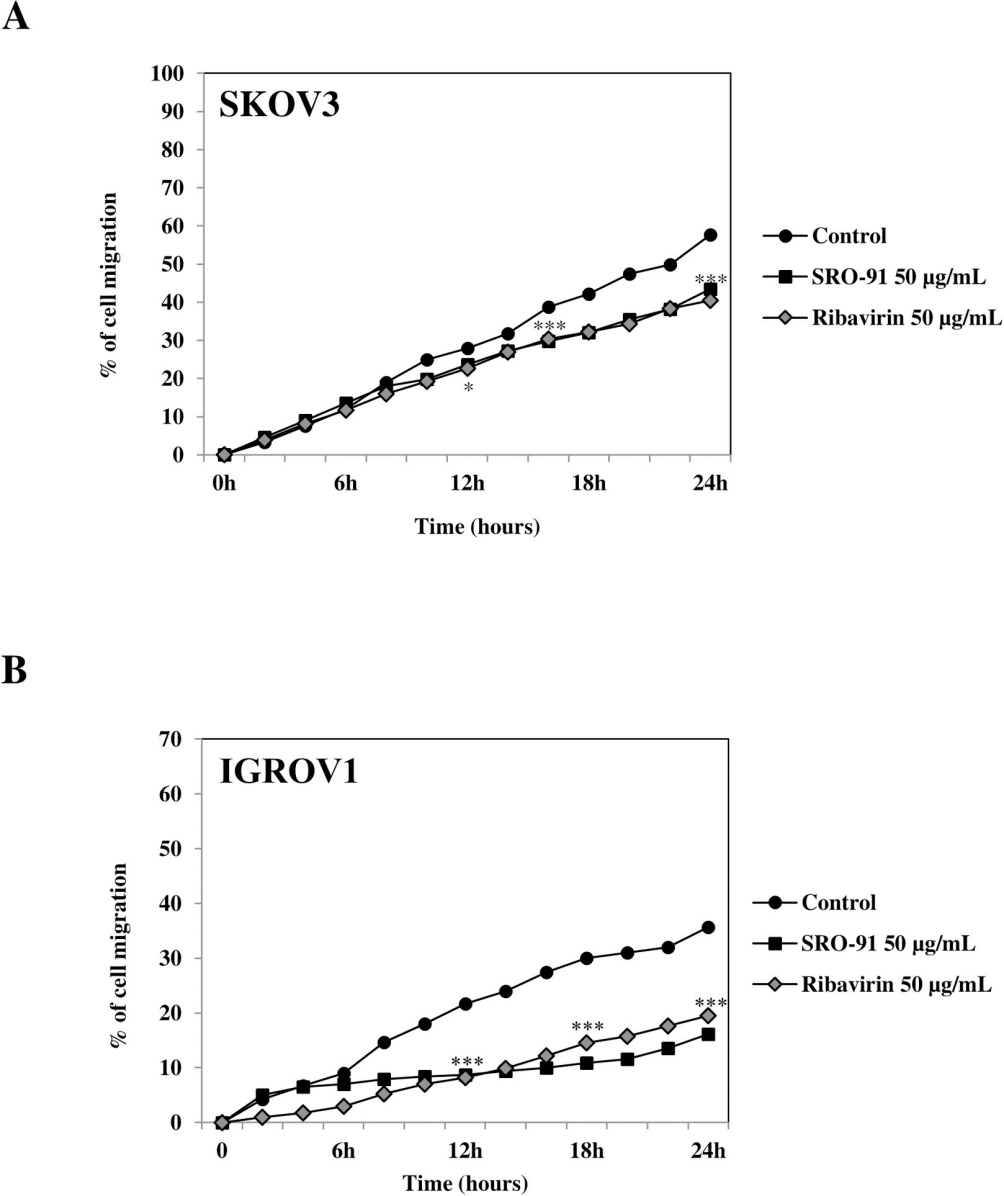
Cancer cells migratory properties after SRO-91 or ribavirin treatment or without treatment (control). **(**A) SKOV3 or (B) IGROV1 cell migration was monitored by time-lapse microscopy over 24 h. Percentage of cell migration in three independent experiments was quantified using ImageJ software. The inhibition of cell migration by SRO-91 or RBV was significant from 12h in both cells lines. P-values were calculated by Paired one-way ANOVA test (*P<0.05, ***P<0.001).

To extend the characterization of the migratory properties of IGROV1 and SKOV3 cells in response to SRO-91 or RBV, we used a quantitative wound healing assay for making measurement of cell migration over 24 h. Cancer cells could migrate into the wounded area without addition of exogenous matrix, indicating that extracellular matrix remnants in the denuded area could sustain migration. To determine whether exogenous RBV or SRO-91 (20 or 50 μg/mL) influenced migration, we found similar effects with the 2 molecules and an inhibition of about 25% in SKOV3 cells and about 40% in IGROV1 cells, (Fig 6).

These data clearly show that RBV and its analog SRO-91 interfere with migration.

### SRO-91 or RBV affects cancer cell colony formation and spheroid organization

**Fig 7:**
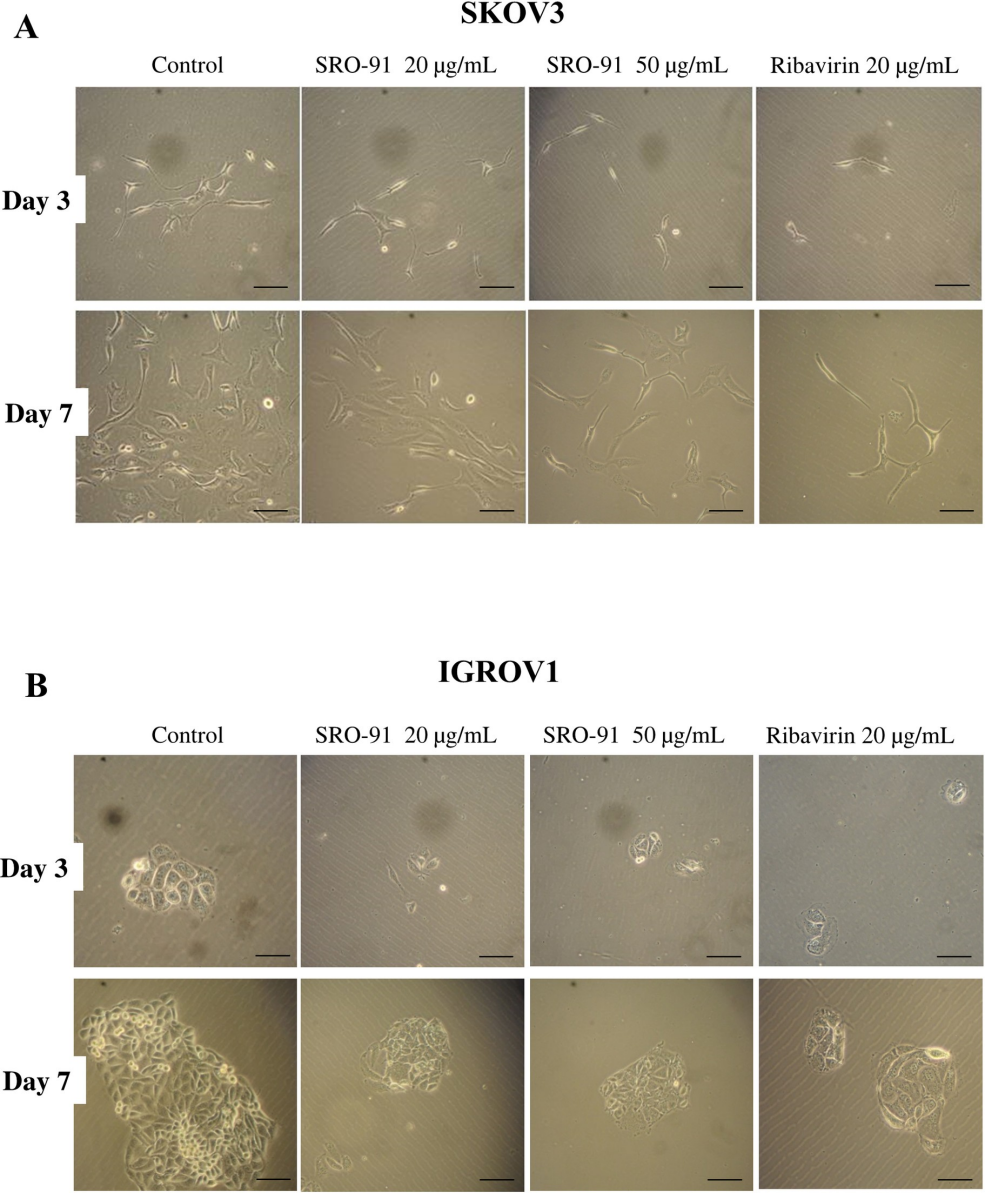
Cancer cells colony forming ability after SRO-91 or ribavirin treatment or without treatment (control). (A) SKOV3 and (B) IGROV1 cancer cells colony formation were followed each day during 7 days by observation of the cells by phase-contrast light microscopy. Pictures (A, B) are representative of at least five fields.

The formation of colonies by SKOV3 and IGROV1 cells in the presence or absence of RBV or SRO-91 was followed by phase-contrast microscopy over 7 days. When cells were seeded under typical conditions, some individual colonies appeared over the time with well-defined outlines for IGROV1 cells (Fig 7B), but were more spread in SKOV3 cells (Fig 7A). In the presence of 20 or 50μg/mL of SRO-91, IGROV1 colonies (representative images are shown in Fig 7B) or SKOV3 plaques were significantly reduced. Similar results were obtained with RBV.

Ovarian carcinoma dissemination is partly due to multicellular aggregate formations (spheroids) that become released within the peritoneal cavity before implantation into the mesothelium. We have studied cancer spheroid formation and established a model of their culture [18,19]. In the present study, we estimated the effect of SRO-91 (or RBV) on IGROV1 spheroid formation using this model of culture conditions in a non-adhesive environment (see Materials and Methods).

**Fig 8:**
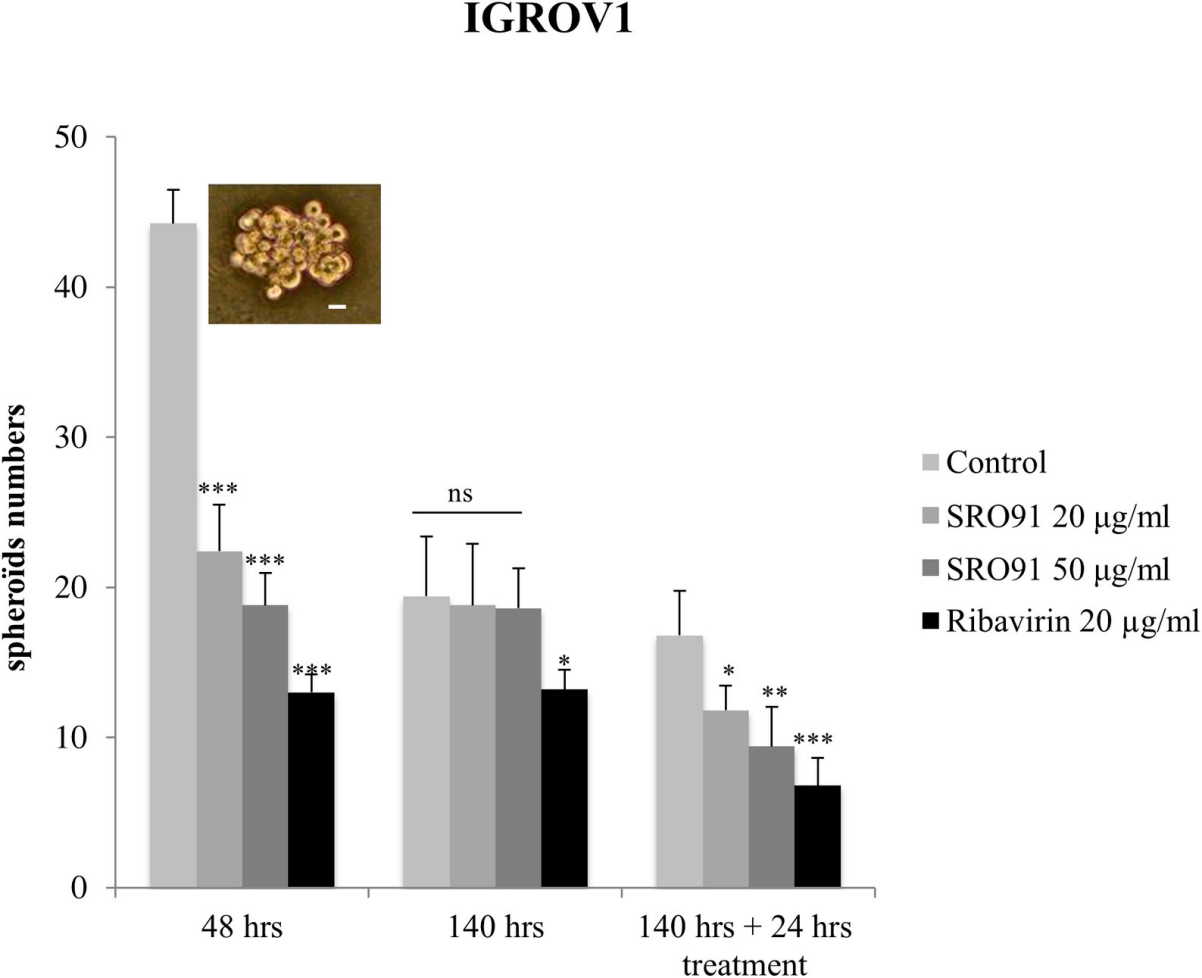
Cancer spheroids formation after SRO-91 or ribavirin treatment or without treatment (control). IGROV1 cancer spheroids formation was followed each day during 7 days. The number of spheroids was counted in at least ten fields in each condition. Inset: IGROV1 spheroid. Scale bar is 50 μm. Results are representative of at least three independent experiments. P-values were calculated by Paired one-way ANOVA test (*P<0.05. **P<0.01, ***P<0.001. ns: no significant).

The number of spheroids was estimated after 48h of culture in the presence or absence of the compounds. SRO-91 and RBV clearly impede spheroids formation (Fig 8). Although this inhibitory effect might have gone on for 140h, renewed addition of the molecules at this time certainly led to further inhibition (Fig 8).

These results suggest that RVB and its analog SRO-91 affect these 2 aggressive phenotypes, i.e. clonogenicity and spheroid formation.

### SRO-91 or RBV acts on the ovarian cancer cells by affecting the localization of eIF4E

To explore the mechanism by which SRO-91 or RBV affects ovarian cancer cell behavior, the localization of eIF4E was determined. Since eIF4E protein is overexpressed in 30% of cancers, it acts as a reference translation initiation factor associated with the translation of genes involved in proliferation, e.g. cyclin D1 or c-myc, stimulating proliferation of cancer cells when overexpressed.

**Fig 9:**
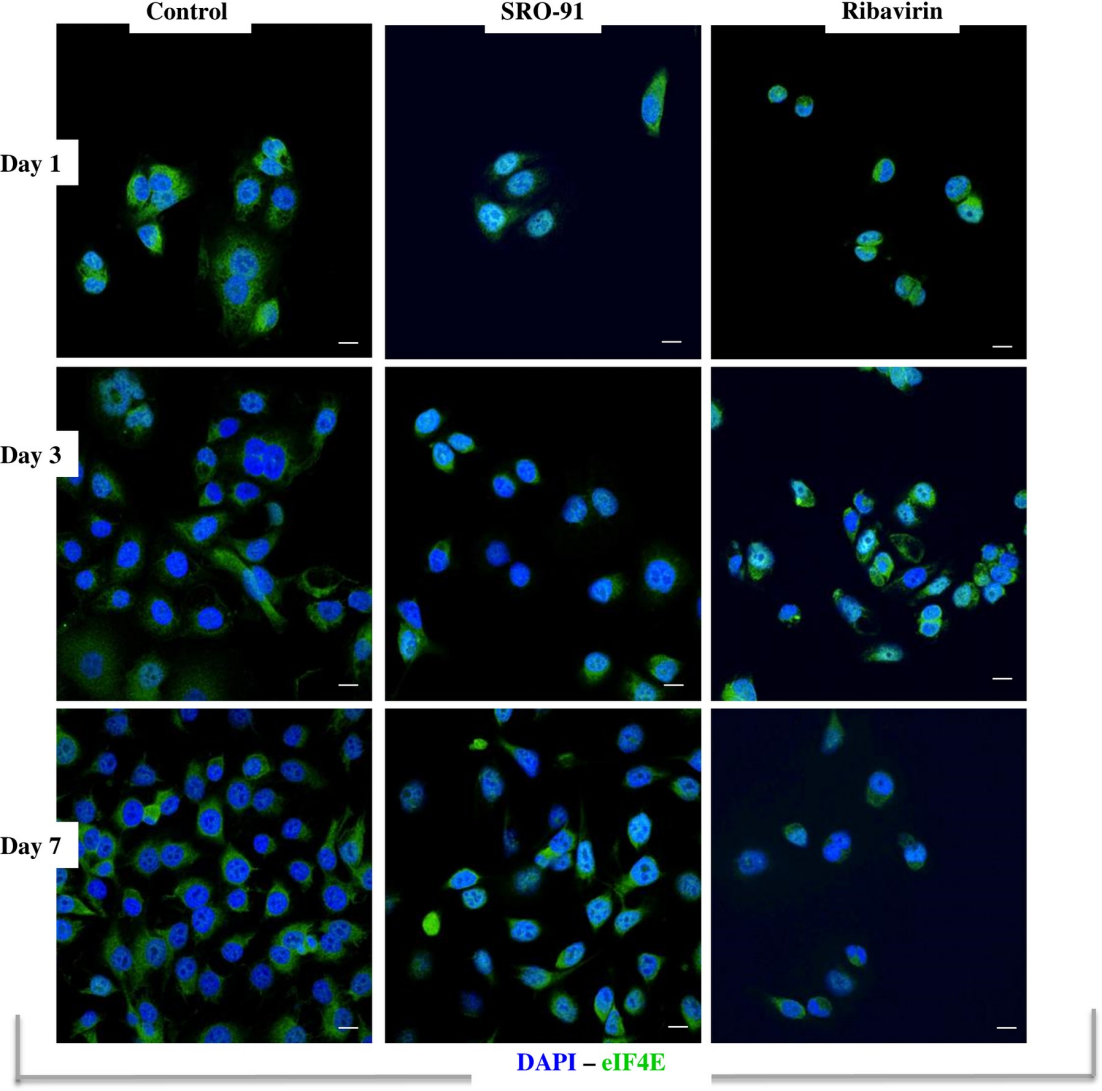
Localization of eIF4E in cancer cells under the effect of SRO-91. Immunofluorescent staining of eIF4E expressed by cancer cells after SRO-91 or ribavirin treatment or without treatment (control) during 7 days of culture. Cell nuclei were stained with DAPI. Staining was examined with laser scanning confocal microscopy. Scale bar is 30 μm.

We wanted to study the localization of eIF4E in ovarian cancer cells under the effect of SRO-91. After seeding IGROV1 cells in the presence or absence of the molecules at 50 μg/mL for 1, 3 and 7 days, immunofluorescence was used to locate the eIF4E. Images of the staining showed diffuse eIF4E in the cytoplasm of untreated cells (Fig 9). In the presence of SRO-91 or RBV, labeling was confined mainly to the perinuclear level, being less diffuse in the cytoplasm with time. IGROV1 cells express eIF4E as soon as day 1, but the SRO-91 treatment confined staining by blocking it at the perinuclear level. Western blotting showed inhibition of eIF4E expression after SRO-91 or RBV treatment (S7 Fig). Although these results need to be improved in more specific analysis into the mechanism of action of this molecule, one hypothesis is that SRO-91 interferes in translation regulation of the genes involved in the proliferation and migration of cancer cells by a system similar to the RBV effect.

### SRO-91 has no cytotoxic effect on non-cancerous cells

Since RBV is an antiviral that can be used in anticancer treatments [5,6], one of the difficulties with it concerns potential side effects, and in particular its toxic effect on the healthy cells. As the ovarian cancer cells preferentially disseminate mainly to the mesothelium of the peritoneum and its underlying connective tissue, the effects of SRO-91 or RBV were estimated on two healthy cell models, fibroblasts (BJ) and mesothelial cells (Met-5A).

**Fig 10:**
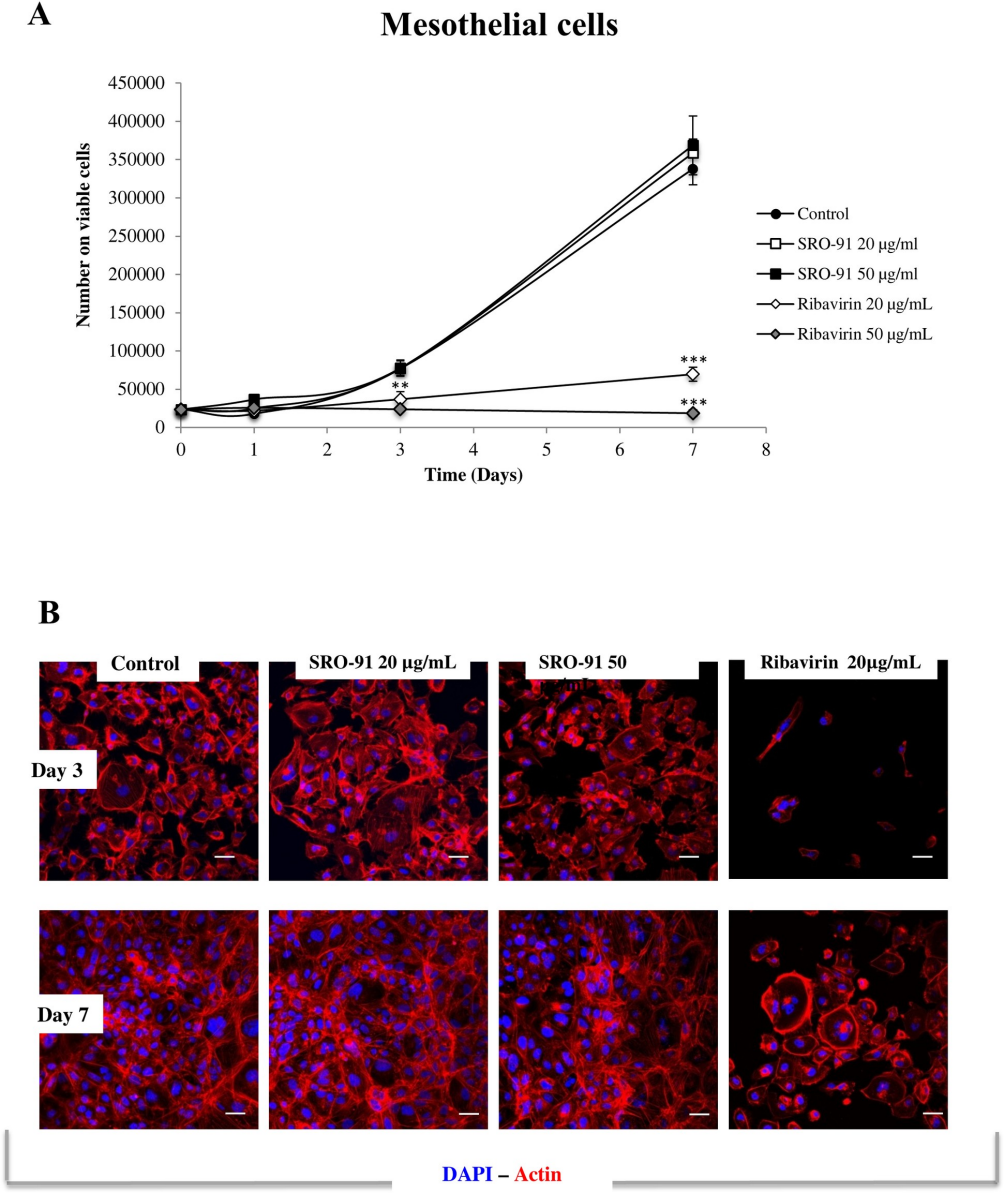
Viability and cell morphology of healthy mesothelial cells after SRO-91 or ribavirin treatment. (A) Mesothelial cells were exposed to ribavirin analog SRO-91 or ribavirin at 0, 20 or 50 μg/mL for 7 days of culture. The number of viable cells was measured daily by the trypan-blue exclusion test. Results are representative of at least three independent experiments performed in triplicate and are expressed as mean ± standard deviation. P-values were calculated by Paired one-way ANOVA test (**P<0.01, ***P<0.001). (B) Immunofluorescent staining of actin cytoskeleton expressed by mesothelial cells after SRO-91 or ribavirin treatment or without treatment (control). Cell nuclei were stained with DAPI. Staining was examined with laser scanning confocal microscopy. Scale bar is 50 μm.

**Fig 11:**
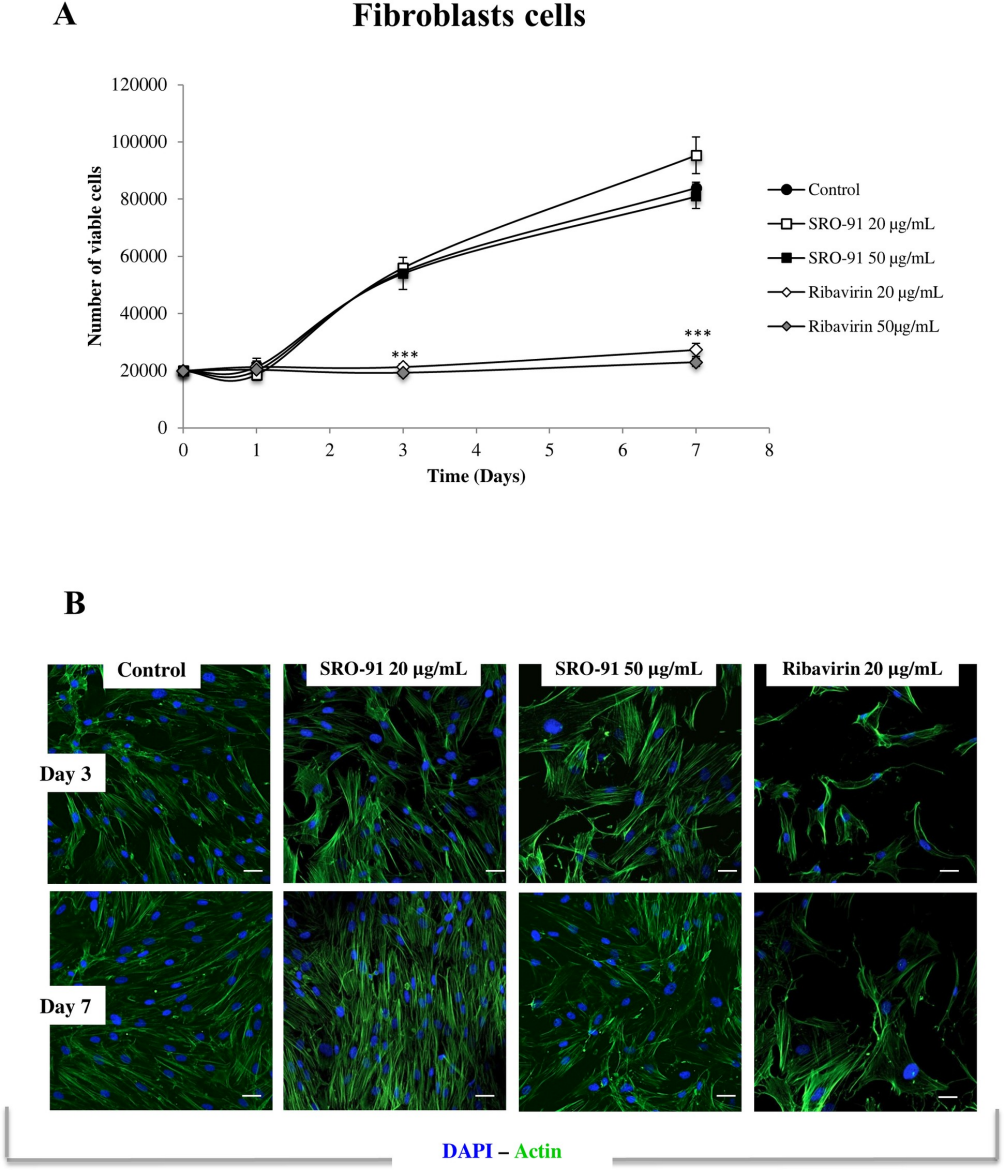
Viability and cell morphology of healthy fibroblasts BJ cells after SRO-91 or ribavirin treatment. (A) Fibroblasts BJ cells were exposed to ribavirin analog SRO-91 or ribavirin at 0, 20 or 50 μg/mL for 7 days of culture. The number of viable cells was measured daily by the trypan-blue exclusion test. Results are representative of at least three independent experiments performed in triplicate and are expressed as mean ± standard deviation. P-values were calculated by Paired one-way ANOVA test (***P<0.001). (B) Immunofluorescent staining of actin cytoskeleton expressed by fibroblasts BJ cells after SRO-91 or ribavirin treatment or without treatment (control). Cell nuclei were stained with DAPI. Staining was examined with laser scanning confocal microscopy. Scale bar is 50 μm.

Whereas RBV drastically inhibited proliferation of mesothelial cells (Met-5A) and fibroblasts (BJ) from the first day of culture, SRO-91 had no effect on the proliferation of their cells (Figs 10A and 11A). The cell morphology and cytoskeleton were markedly modified following treatment with RBV, but SRO-91 had no significant effect (Figs 10B and 11B).

These results demonstrate that SRO-91 does not affect morphology, viability and proliferation of normal cells compared to RBV, indicating that SRO-91 is preferentially toxic to cancer cells compared to normal cells; as such it could be of interest in the treatment of ovarian cancer dissemination, particularly if it worked in combination with anti-cancer drugs such as cisplatin.

### SRO-91 or RBV inhibits the implantation of cancer cells into the peritoneal mesothelium

**Fig 12:**
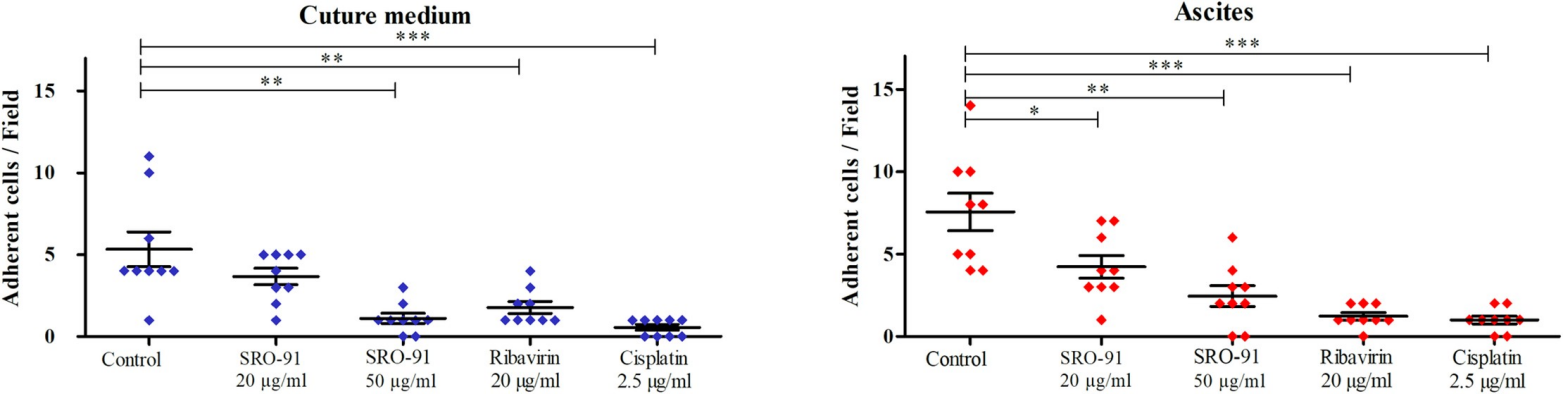
Implantation of cancer cells into the peritoneal mesothelium after SRO-91 or ribavirin treatment. Co-cultures of mesothelial cells grown as monolayer and DiI-labeled tumor cells were observed using a Nomarski filter to ensure mesothelial integrity. Cancer cell adhesion to the mesothelial monolayer was determined by counting fluorescent cells on the underlying non-fluorescent mesothelial cells with the software ImageJ ®. P-values were calculated by the unpaired t-test was performed (*** P<0.001, ** P<0.01, * P<0.05).

Following implantation into the peritoneal mesothelial surface, ascites accumulates, which acts as a reservoir of nutrients and cellular components, potentiating metastasis and leading to a poorer prognosis. The ability of cancer cells to attach to the mesothelium in the presence of RBV, SRO-91 or cisplatin (gold standard drug in the treatment of ovarian cancer) in culture medium (a FCS free buffer) or in the ascitic tumor microenvironment (from patients, see Materials and Methods) was measured. A suitable model of co-culture, previously described by our team [21], was used to assess IGROV1 ovarian cancer cell adhesion to healthy Met-5A mesothelial cells. Cancer cells were directly allowed to adhere for 1h to confluent mesothelial cells in FCS-free culture medium (to avoid serum protein interference in the adhesion process and estimate solely the effect of treatments on this adhesion process), or in ascitic fluid to get closer to the physiopathological conditions of peritoneal implantation. In both models, SRO-91 significantly inhibited cancer cell attachment to the mesothelium, as did RBV or cisplatin (Fig 12). These findings suggest that SRO-91 might be combined with cisplatin as a promising protocol for ovarian cancer treatment.

## Discussion

Among new compounds, such as RBV, have been proposed, although RBV is used in the treatment of hepatitis C infections and more recently some cancers. However, it is not without many side effects, suggesting the synthesis of analogs might be better. SRO-91, an analog of RBV, has therefore been explored regarding the behavior of ovarian carcinoma and normal cells compared with RBV.

A dose- and time-dependent inhibitory effect of SRO-91 (a more stable nucleoside due to the presence of a C-C bond making it more resistant and insensitive to glycosidases) is shown on the proliferation of SKOV3 and IGROV1 cells, in comparison with RBV, at clinically relevant concentrations. Whereas for SKOV3 cells inhibition of proliferation increased up to the 7th day of culture, this effect tends to ease off for IGROV1 cells. “Resumption” of the proliferation in the latter cell type could, on the one hand, be due to the cell cycle being shorter than that of SKOV3 cells. The time of doubling of SKOV3 cells is ~30 h, while that of the IGROV1 is ~20 h which would allow IGROV1 cells to metabolize and thus deplete the medium faster in SRO-91. On the other hand, this effect might be due to the ability of IGROV1 cells to form spheroids that have more resistance to chemotherapeutics. Indeed, these 2 ovarian cell lines could make interesting subjects for drug exposure because: (i) IGROV1 cells can form free-floating clusters in culture, whereas SKOV3 derived from ascites gave only isolated cells; (ii) they differ in their response to cisplatin [18, 22]. RBV-dependent dose-time inhibitory effects on the proliferation of breast cancer cells (MCF-7, SKBr3, BT474) and colon cancer cells (HCT-116, SW620) using a colorimetric method [9,23] or ovarian (OVCAR-5), hepatic (HepG2) and leukemic (K562) cancer lines, by trypan blue counting have been reported [7]. Variability of the responses depending on the heterogeneity of cancers may be involved, which in some way might be related to the initial level of expression of eIF4E protein in cancer cells [23–26].

We also focused our attention on other features involved in cancer dissemination—migration, clonogenicity and spheroid formation—in response to SRO-91 or RBV treatment. An inhibitory effect of SRO-91 or RBV on all these events was found. Thiazofurin, another analog of RBV, also inhibited migration of mouse lung cancer cells and human melanoma cells [27]. Migrating cells tend to have more pseudopodia, which can be dependent on signaling involving, e.g., RhoA and ErbB2 [28]. Inhibition of cell migration by SRO-91 could therefore be due to an effect on one or more molecules of these signaling pathways, especially as it is also involved in cell adhesion. Unlike Thiazofurin [27], SRO-91 does not seem to affect the adhesion of cancer cells (S4 Fig).

The effects of SRO-91 or RBV on cell and nuclear morphology, on the cytoskeleton, and on the localization of extracellular matrix-receptor αv integrins have been explored. αv integrins and their extracellular matrix and cytoskeleton partners seem to have a central role in the behavior of ovarian cancer cells [19,21,29–31]. Thus, staining of nuclear DNA, actin and αv integrins showed that SRO-91, like RBV, has significantly increased cell and nuclear volume with slight impact on adhesion systems (rearrangement of focal adhesions) and cell morphology (actin organization), probably associated to a decrease of cell density and an effect on cell migration. This was confirmed by adhesion assays in the presence of SRO-91, which does not affect adhesion of cancer cells (S4 Fig). Moreover, staining of extracellular matrix molecules, vitronectin -main αv integrins receptor-, and laminin, shows that SRO-91, like RBV, does not act on the organization of the extracellular matrix and matrix microenvironment (S5 Fig) of cancer cells–an effect of RBV on several other cell models [7,24,32]—by its specific action on eIF4E.

The potential use of RBV analog on the dissemination of ovarian cancer cells requires more knowledge of the mechanism. However, we wanted to determine whether inhibition of proliferation was associated with cell death by analyzing the expression of the PARP cleaved protein, a late effector of apoptosis. SRO-91 does not cause PARP cleavage, which may reflect either a lack of activation of the apoptotic pathway or the activation of an effector upstream of this pathway that can modulate the cleavage of PARP. In IGROV1 cells, slight cleavage occurs after 3 days of culture, probably due to the formation of non-adherent multicellular aggregates [20]. In all cases, the inhibition of proliferation due by SRO-91 is not explicable by cell death through apoptosis as confirmed by cell cycle results. No apoptosis was observed for endothelial cells (HUVECs) treated with RBV, inhibition of proliferation being attributed to G0/G1 arrest [33]. Other studies suggest that the arrest by RBV in colon cancer cells is at the G2/M transition [23]. Our BrdU incorporation tests showed a decrease in the number of cells able to incorporate this analog, but no apparent blockage in the phases of the cell cycle, particularly S-phase (S3 Fig). These results confirm the decrease in the overall cell density upon treatment which is consistent with the slowdown of cell cycle progression, increased doubling time and inhibition of proliferation in both cell lines. The effect of bioactive molecules like TGF-beta was suggested to increase the doubling time of cells without significantly affecting the cell-cycle distribution during few days [34]. Recent study reports that when DNA damage occurred, in response to stress, DNA synthesis continues but the overall rate of S phase progression is slowed [35].

Our results show a significantly increased of nuclear area and cell shape with treatment. The growth of the nuclear volume is not correlated with the beginning of the DNA synthesis. The nuclear volume starts to increase already 6 hr prior DNA synthesis [36]. Cell shape could affect nuclear volume through a link with cell volume, as has been reported for other cell types. Relationship between cell and nuclear size and the cell cycle remains controversial [37]. Nuclear volume could be controlled by changes in biochemical signaling downstream of the ECM and growth factors given by cell spreading. Thus, increase of cell spreading and nuclei area could be explained by chromatin decondensation [37] without apparent correlation with DNA synthesis as shown in our study. This hypothesis would require more exploration.

In this study, SRO-91 effects are similar to those obtained with RBV, which coincides with its role on many genes involved in regulation of expression of proteins with important functions in tumor progression, notably inhibition of one of them, i.e. eIF4E. eIF4E is frequently overexpressed in cancers—leukemic, breast, ovarian, esophageal, colon and cervical [5–7,9,23–26]. It affects the expression of genes involved in the cell cycle, proliferation, invasion and angiogenesis (e.g. Cyclin D1 / E1 / B1 / NBS1 / C-Myc, VEGF) by increasing their mRNAs in the cytoplasm and therefore their translation [8,9,11]. Thus, RBV acts on eIF4E, preventing it from acting as an initiator of translation. We found a similar inhibitory effect of the SRO-91 on the localization of eIF4E, confining it to a perinuclear location, where it might interfere with translation regulation of the molecules involved in the proliferation and survival of cancer cells in the presence of the SRO-91.

Few studies have investigated the cytotoxicity of RBV on non-cancerous cells, although it has been associated with cytotoxic effects [23,38]. We compared the cytotoxicity of SRO-91 and RBV on normal mesothelial cells or fibroblasts BJ at clinical concentrations. Although RBV appears to be highly toxic to Met-5A and BJ cells, SRO-91 was not. Thus the possible use of SRO-91 as anti-cancer treatments is enhanced by its preferential cytotoxic action on cancer cells, although whether this is true at higher concentration needs to be considered.

We finally compared the effects of SRO-91, RBV or Cisplatin on peritoneal implantation within an ascitic microenvironment more related to *in vivo* pathology. SRO-91 inhibited the implantation of cancer cells on Met-5A cells under classical or ascitic conditions in the same way to RBV and Cisplatin. Ascitic fluid in the peritoneal cavity seems to promote cell migration or the formation of spheroids by ovarian cancer cells [18]. The formation of spheroids is characterized by the epithelio-mesenchymal transition. Epithelial-type cells have many adherent junctions, desmosomes and tight junctions, whereas mesenchymal cells are isolated from each other and have exacerbated migration and invasive properties [39]. Incubation of cells in ascitic fluid may trigger this epithelial-mesenchymal transition, which needs an in-depth study because of the chemoresistance of cancer cells (responsible for recurrences) that has been associated with TEM [40,41]. These considerations suggest the possibility of using of SRO-91 in combination with cisplatin in cancer therapy.

## Conclusions

The effects of SRO-91, an analog of RBV, on the behavior of ovarian carcinoma cells have been explored and compared to RBV. Inhibitory effects of SRO-91, similar to those of RBV, were found on the different aspects of cell dissemination, including proliferation, migration, clonogenicity, spheroid formation and peritoneal implantation. The relevance to the use of SRO-91 as an anticancer treatment is reinforced it relative non-toxicity to non-cancerous cells that were tested. The mechanism of entry of SRO-91 to cells and to its site of action needs to be more thoroughly investigated. Thus, these results indicate the potential use of SRO-91 in anticancer therapy, with the added possibility of some synergism with other chemotherapeutics agents in combination therapy for ovarian and possibly other types of cancer.

## Supporting information

S1 Fig Uncropped blots for Fig 3A. Scan Blot of PARP / actin in presence of SRO-91. MW: Molecular Weight (kDa). Capture image was acquired by densitometer (Biorad).
(TIF)

S2 Fig Uncropped blots for Fig 3B. Scan Blot of PARP / actin in presence of SRO-91. MW: Molecular Weight (kDa). Capture image was acquired by densitometer (Biorad).
(TIF)

S3 Fig Cell cycle analysis of SKOV3 and IGROV1 cells treated with SRO-31 or RBV. Representative flow cytometric analysis for DNA content in ovarian cancer cells treated with 50 μg/ml of SRO-91 or RBV during 3 and 7 days. The percentage of cell cycle distribution in Sub-G1, Go/G1, S and G2/M phases were determined with Expo32 acquisition software (Beckman Coulter).
(TIF)

S4 Fig Ovarian cancer cell adhesion assay after SRO-91 treatment. Adhesion of SKOV3 and IGROV1 cells were examined on a coating of fibronectin plasma protein (10μg/ml) and treated with 0 to 50 μg/ml of SRO-91. After 2 hours, adherent cells were revealed by cristal violet coloration and absorbance was read at 595nm. Values are expressed as mean ± SD. Data represent means of three independent experiments done in triplicates.
(TIF)

S5 Fig Extracellular matrix proteins organization in treated ovarian cancer cells. Immunofluorescent staining of vitronectin and laminin expressed by IGROV1 cells after SRO-91 or ribavirin treatment (50 μg/ml) or without treatment (control). Cell nuclei were stained with DAPI. Staining was examined with laser scanning confocal microscopy. Scale bar is 50 μm.
(TIF)

S6 Fig Relative cell size and nuclear volume of ovarian cancer cells after SRO-91 treatment. Representative flow cytometric analysis for DNA content (nuclear shape) and the forward scatter (FS) parameter. The nuclear area was determined with Expo32 acquisition software (Beckman Coulter).
(TIF)

S7 Fig Uncropped blots for eIF4E expression. Representative Western blots for eIF4E in ovarian cancer cells treated with 50 μg/ml SRO-91 or RBV or without treatment (control). Tubulin was used as a loading control. MW: Molecular Weight (kDa). Capture image was acquired by densitometer (Biorad).
(TIF)
