# Supplementary figures and images for: Evaluation of the potential of a new ribavirin analog impairing the dissemination of ovarian cancer cells
Source: PLoS One. 2019 Dec 11;14(12):e0225860. doi: 10.1371/journal.pone.0225860 (PMC6905583; doi:10.1371/journal.pone.0225860)

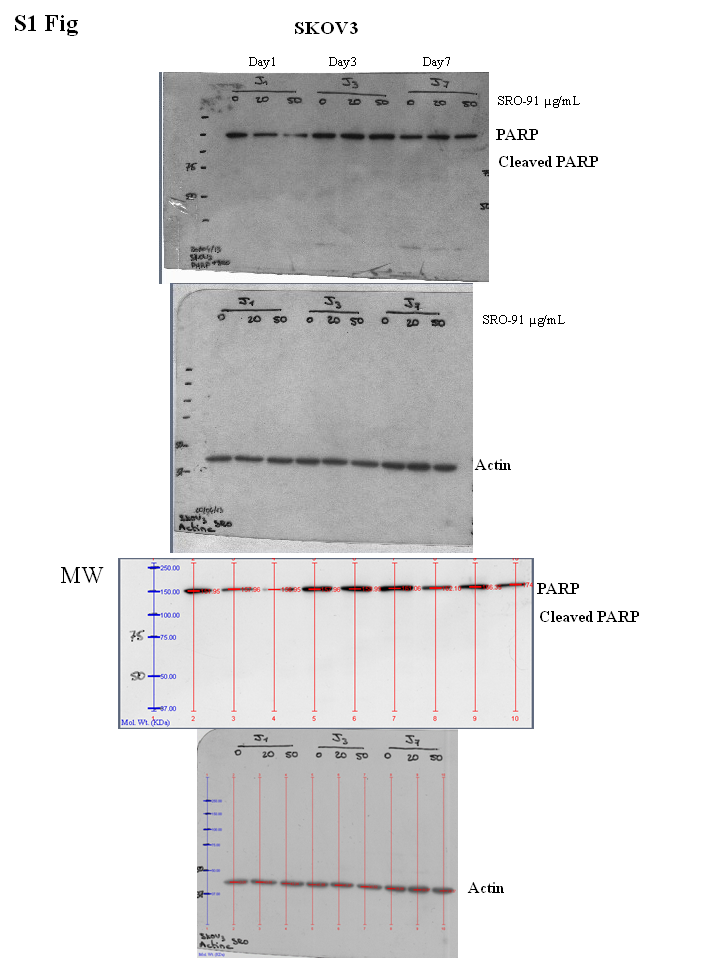

Supplement: S1 Fig — Scan Blot of PARP / actin in presence of SRO-91. MW: Molecular Weight (kDa). Capture image was acquired by densitometer (Biorad). (TIF) [file pone.0225860.s001.TIF]

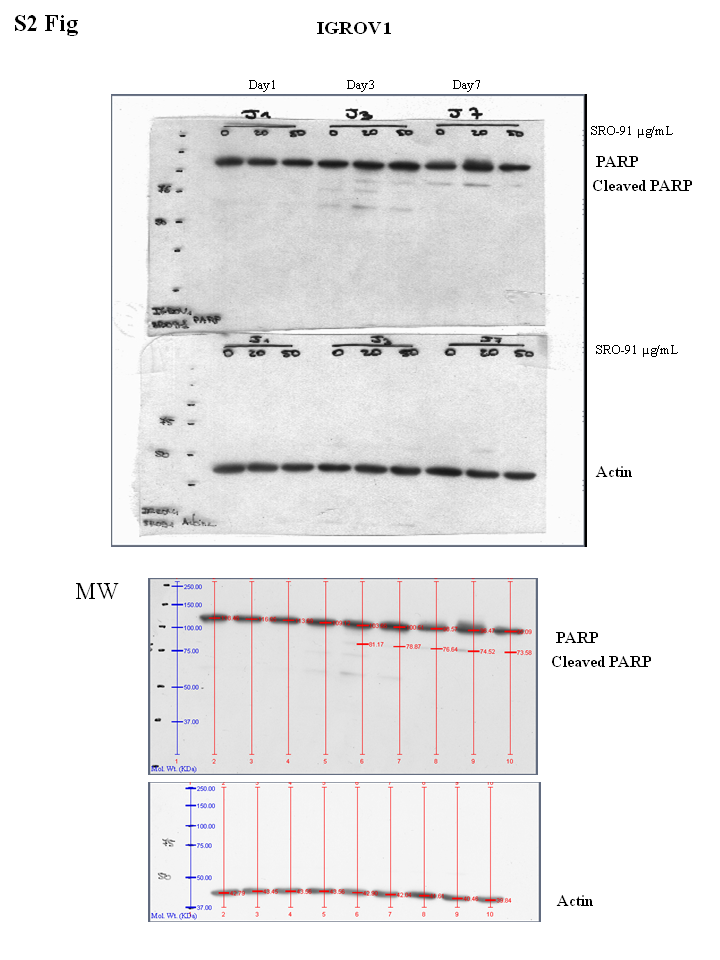

Supplement: S2 Fig — Scan Blot of PARP / actin in presence of SRO-91. MW: Molecular Weight (kDa). Capture image was acquired by densitometer (Biorad). (TIF) [file pone.0225860.s002.TIF]

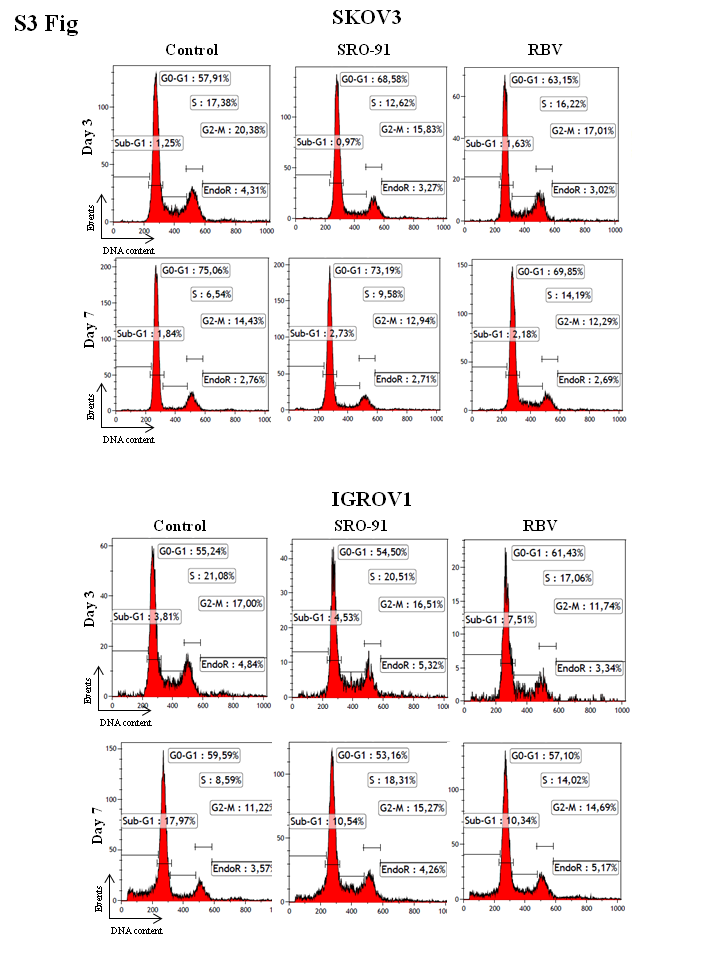

Supplement: S3 Fig — Representative flow cytometric analysis for DNA content in ovarian cancer cells treated with 50 μg/ml of SRO-91 or RBV during 3 and 7 days. The percentage of cell cycle distribution in Sub-G1, Go/G1, S and G2/M phases were determined with Expo32 acquisition software (Beckman Coulter). (TIF) [file pone.0225860.s003.TIF]

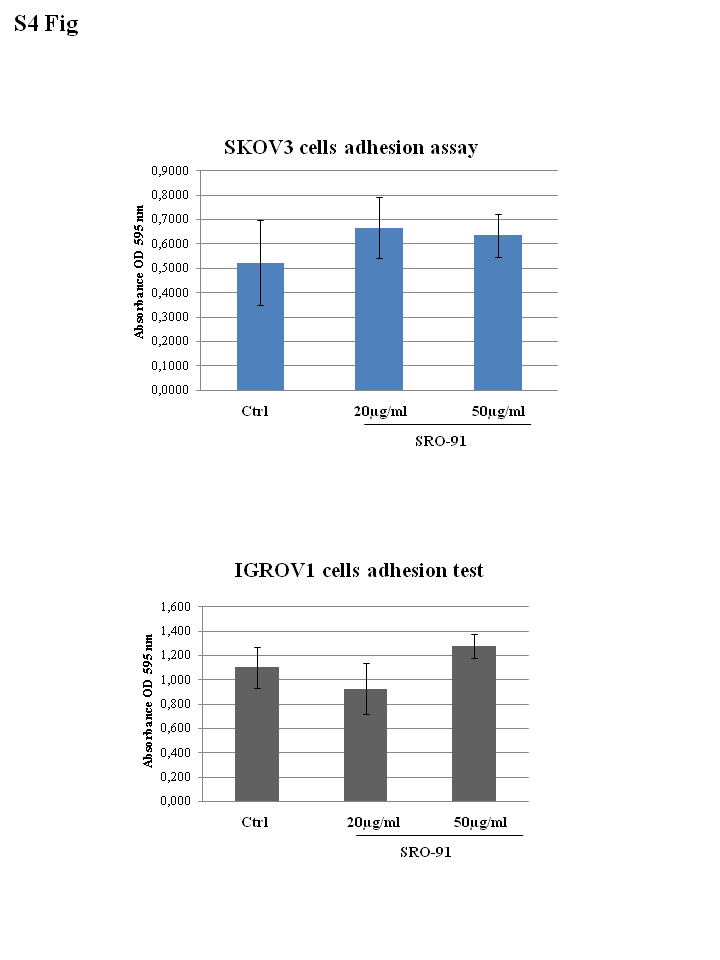

Supplement: S4 Fig — Adhesion of SKOV3 and IGROV1 cells were examined on a coating of fibronectin plasma protein (10μg/ml) and treated with 0 to 50 μg/ml of SRO-91. After 2 hours, adherent cells were revealed by cristal violet coloration and absorbance was read at 595nm. Values are expressed as mean ± SD. Data represent means of three independent experiments done in triplicates. (TIF) [file pone.0225860.s004.TIF]

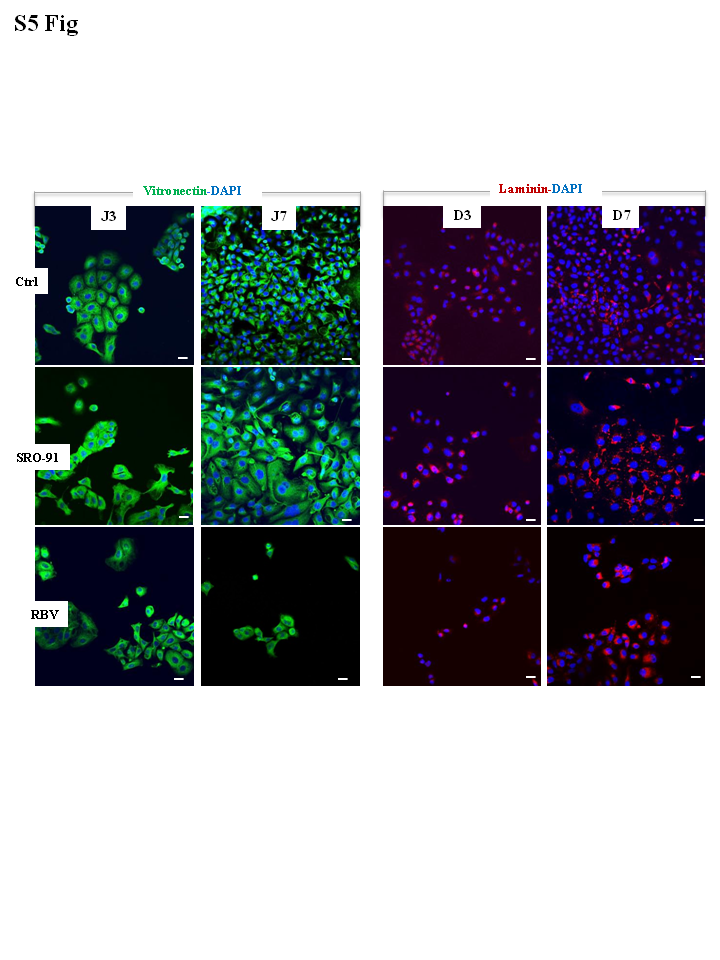

Supplement: S5 Fig — Immunofluorescent staining of vitronectin and laminin expressed by IGROV1 cells after SRO-91 or ribavirin treatment (50 μg/ml) or without treatment (control). Cell nuclei were stained with DAPI. Staining was examined with laser scanning confocal microscopy. Scale bar is 50 μm. (TIF) [file pone.0225860.s005.TIF]

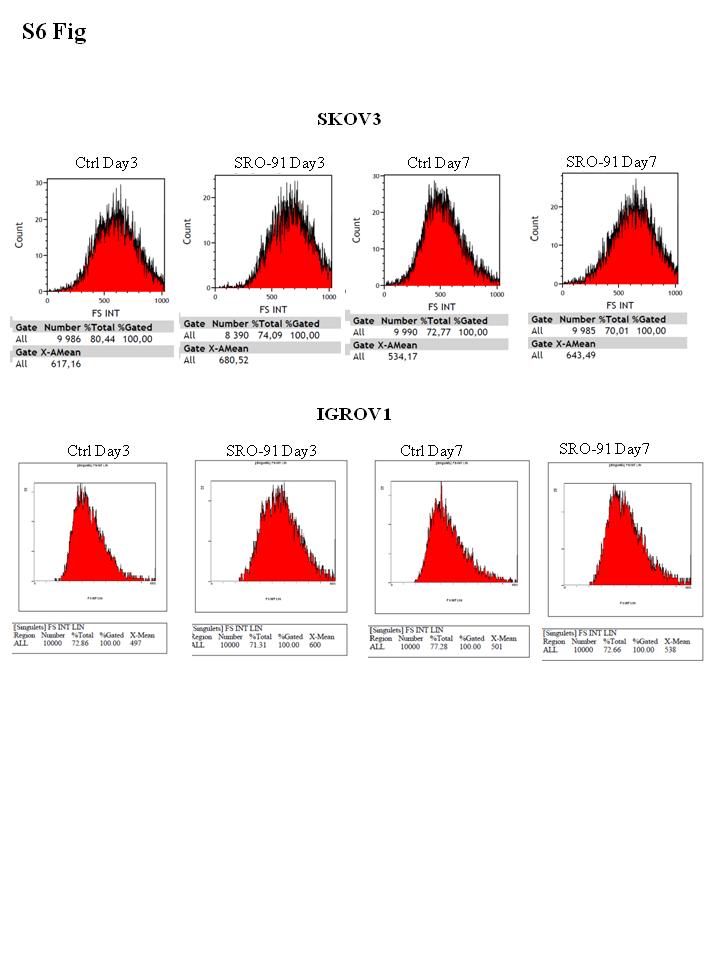

Supplement: S6 Fig — Representative flow cytometric analysis for DNA content (nuclear shape) and the forward scatter (FS) parameter. The nuclear area was determined with Expo32 acquisition software (Beckman Coulter). (TIF) [file pone.0225860.s006.TIF]

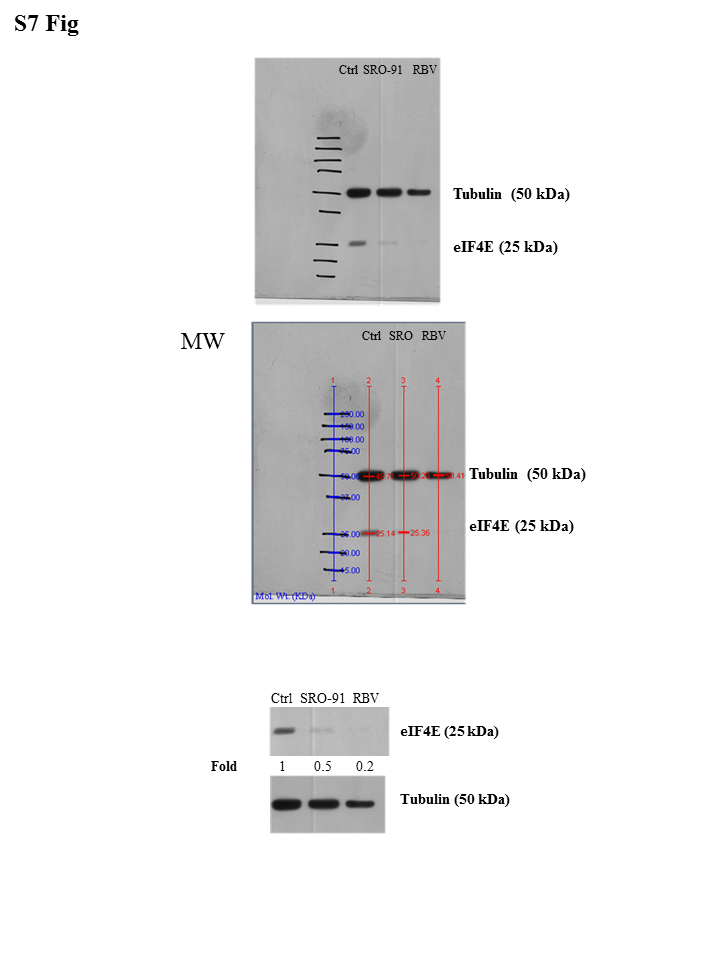

Supplement: S7 Fig — Representative Western blots for eIF4E in ovarian cancer cells treated with 50 μg/ml SRO-91 or RBV or without treatment (control). Tubulin was used as a loading control. MW: Molecular Weight (kDa). Capture image was acquired by densitometer (Biorad). (TIF) [file pone.0225860.s007.tif]
